# Colonic Dopaminergic Neurons Changed Reversely With Those in the Midbrain via Gut Microbiota-Mediated Autophagy in a Chronic Parkinson’s Disease Mice Model

**DOI:** 10.3389/fnagi.2021.649627

**Published:** 2021-04-12

**Authors:** Xin Liu, Zhong-Rui Du, Xiong Wang, Kar-Him Luk, Cheuk-Hin Chan, Xu Cao, Qing Zhao, Fang Zhao, Wing-Tak Wong, Ka-Hing Wong, Xiao-Li Dong

**Affiliations:** ^1^Key Laboratory of Food Biological Safety Control, The Hong Kong Polytechnic University Shenzhen Institute, Shenzhen, China; ^2^Research Institute for Future Food, The Hong Kong Polytechnic University, Hong Kong, China; ^3^Department of Applied Biology and Chemical Technology, The Hong Kong Polytechnic University, Hong Kong, China; ^4^College of Physical Education, Ludong University, Yantai, China; ^5^Department of Neurology, Shenzhen University General Hospital, Shenzhen University Clinical Medical Academy, Shenzhen, China; ^6^Department of Neurology, Shenzhen People’s Hospital, Jinan University, Shenzhen, China; ^7^Department of Neurology, Linzi Maternal and Child Health Hospital of Zibo, Zibo, China; ^8^Beijing Genomics Institute (BGI)-Qingdao, BGI-Shenzhen, Qingdao, China

**Keywords:** Parkinson’s disease, dopamine, tyrosine hydroxylase, autophagy, apoptosis, short chain fatty acids, gut microbiota dysbiosis

## Abstract

The role of gut-brain axis in the pathogenesis of Parkinson’s disease (PD) have become a research hotspot, appropriate animal model to study gut-brain axis in PD is yet to be confirmed. Our study employed a classical PD mice model achieved by chronic MPTP (1-methyl-4-phenyl-1,2,3,6-tetrahydropyridine) injection to study concurrent changes of dopaminergic neurons in the midbrain and the colon of mice. Our results showed such a PD model exhibited apparent locomotor deficits but not gastrointestinal dysfunction. Tyrosine hydroxylase expressions and dopamine content reduced greatly in the substantia nigra pars compacta (SNpc) or striatum, but increased in the colon of PD mice. Mechanism investigation indicated autophagy activity and apoptosis were stimulated in the SNpc, but inhibited in the colon of PD mice. Interplay of gut microbiota (GM) and autophagy in response to chronic MPTP injection led to GM dysbiosis and defective autophagy in mice colon. Meanwhile, fecal short chain fatty acids (SCFAs), acetate and propionate in particular, declined greatly in PD mice, which could be attributed to the decreased bacteria abundance of phylum *Bacteroidetes*, but increased abundance of phylum *Firmicutes*. GM dysbiosis derived fecal SCFAs might be one of the mediators of downregulated autophagy in the colon of PD mice. In conclusion, colonic dopaminergic neurons changed in the opposition direction with those in the midbrain via GM dysbiosis-mediated autophagy inhibition followed by suppressed apoptosis in response to chronic MPTP injection. Such a chronic PD mice model might not be an ideal model to study role of gut-brain axis in PD progression.

## Introduction

The pathological hallmarks of Parkinson’s disease (PD) is the slow and progressive loss of dopaminergic neurons in the nigrostriatal pathway, which leads to classical motor symptoms like bradykinesia, rigidity, and resting tremor (Seppi et al., 2019). Meanwhile, impairment of dopaminergic neurons in the enteric nervous system (ENS) of the intestine was found to contribute to reduced intestinal motility in PD patients (Nadeau et al., 2019). Gastrointestinal (GI) dysfunction is the major non-motor manifestation at the early stage of PD, and it occurs long before the appearance of motor symptoms (Yang et al., 2019). The GI tract, ENS, gut microbiota (GM) and gut-brain crosstalk have recently become research hotspots. The pathological process of the gut-brain axis might spread from the gut to the brain and contribute to the pathogenesis of PD (Mulak and Bonaz, 2015), but the underlying mechanisms are not fully understood.

Apoptosis and autophagy are two major machineries of the degeneration of dopaminergic neurons in the pathogenesis of PD (Ghavami et al., 2014). The balance between apoptosis and autophagy is vital for maintaining normal cellular homeostasis, and their imbalance accelerates neurodegeneration and is closely related to the progression of PD (Liu et al., 2019). Excessive autophagy, which simultaneously accelerated apoptosis, leading to quick death and loss of dopaminergic neurons (Li et al., 2011). Recent studies demonstrated GM is closely linked to intestinal pathology and inflammation through its interplay with autophagy in mechanism studies of colon cancer and inflammatory bowel disease (IBD). GM dysbiosis could regulate autophagy (Kim et al., 2020) and autophagy has a role in the control of GM compositions (Larabi et al., 2020). Many clinical studies observed GM dysbiosis in PD patients, which plays key roles in aggravating PD through the promotion of inflammatory cascades or oxidative stress in the brain via short chain fatty acids (SCFA)-production or a lipopolysaccharide (LPS)-mediated mechanism (Breen et al., 2019; Roy Sarkar and Banerjee, 2019; Yang et al., 2019; Pascale et al., 2020). However, little researches have investigated the relationships of GM dysbiosis with autophagy and apoptosis in the intestine during PD pathogenesis.

Researchers are actively seeking for an appropriate animal model to study the gut-brain axis in PD progression. Although many classical PD mice or rats models which can mimic brain pathology of patients are applicable, it is still unknown if intestinal pathology in these models can mimic those occurred in PD patients and correspond to brain pathology. MPTP (1-methyl-4-phenyl-1,2,3,6-tetrahydropyridine), a selective dopamine (DA) neuron toxin, is the most widely used toxin to generate PD animal models, and can represent the earliest phase of PD (Meredith and Rademacher, 2011). Different protocols to use MPTP are applied in making PD animal model, among which, acute (1 day), subacute (5 days), and chronic (5 weeks) intraperitoneal (i.p.) injection of MPTP to mice are three classical usages. These three classical MPTP mice models can show obvious behavioral deficits and dopaminergic neuronal loss in the midbrain. MPTP impairs dopaminergic neurons not only in the central nervous system (CNS), but also in the ENS. Recent studies (Ellett et al., 2016; Poirier et al., 2016; Lai et al., 2018; Sun et al., 2018; Zhou et al., 2019; Dong et al., 2020; Liao et al., 2020) have observed changes of GM composition, GM metabolites of short chain fatty acids (SCFAs), and/or enteric dopaminergic neurons in these three classical MPTP mice models. The detailed information is summarized and shown in Supplementary Table 1. It was found different MPTP injection protocols and even different collection time of animal samples after MPTP injection could lead to various alterations of GM compositions and SCFAs production (Supplementary Table 1). Two studies (Ellett et al., 2016; Poirier et al., 2016) discovered that acute injection of MPTP in 1 day led to loss of dopaminergic neurons in the ileum by immunohistochemistry staining, but no GM and SCFAs data were provided in their studies. Only one published study (Lai et al., 2018) using chronic MPTP injection protocol (twice a week for 5 weeks) reported significant changes of GM compositions and dopaminergic neurons in the ileum when animal tissue samples were collected on the 2nd day and 22nd day, but not at the end of experiment (classically 5 weeks). No published paper has observed simultaneous alterations of GM compositions, SCFAs together with enteric dopaminergic neurons and explored their relationships with autophagy and apoptosis in ENS in either MPTP mice model.

In this study, we chose a classical PD mice model made by chronic MPTP injection (twice a week for 5 weeks) and collected animal samples after 5 weeks, to study the concurrent changes of dopaminergic neurons in the midbrain and colon, followed by mechanism investigations to reveal the relationships of enteric dopaminergic neurons with autophagy, apoptosis and GM. Our study purpose is to explore if intestinal pathology in such a chronic MPTP model can mimic those occurred in PD patients and correspond to brain pathology, and this study will provide evidence to appraise if it could be an appropriate animal model to study the role of gut-brain axis in PD progression.

## Materials and Methods

### Animal Experimental Design

**FIGURE 1:**
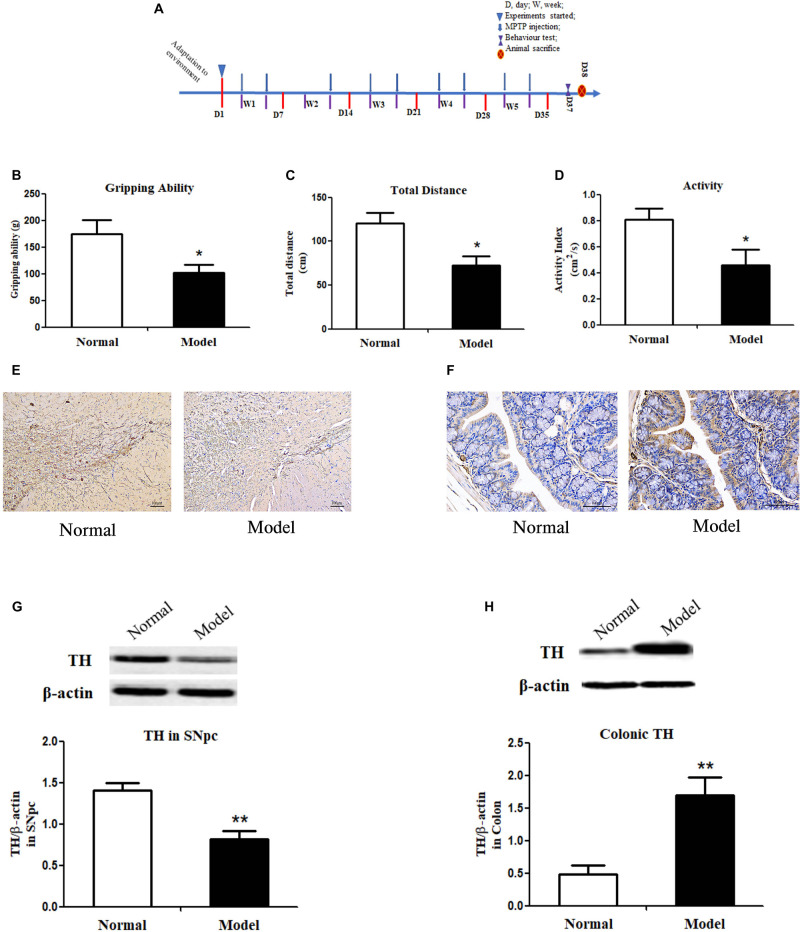
Experimental timeline, behavior tests, immunochemistry staining of tyrosine hydroxylase and its protein expressions in both the substantia nigra pars compacta and colon of mice. **(A)** Timeline of animal experiments. **(B)** Grip strength test of mice indicated by gripping ability (g); Open field test of mice indicated by total distance traveled (cm) **(C)** and activity index (cm^2^/s) **(D)** within a 5-min period (*n* = 8). Representative IHC staining of TH in sections of the SNpc **(E)** and the colon **(F)** in normal and PD mice. TH protein expressions in the SNpc **(G)** and the colon **(H)** of normal and PD model mice (One representative band for TH and endogenous β-actin from each group; and the following bar chart (*n* = 6–8) shows the ratio of TH/β-actin in the SNpc and colon of mice). Values are expressed as mean ± SEM. *p < 0.05, **p < 0.01 vs. Model group. IHC, immunochemistry; TH, tyrosine hydroxylase; SNpc, substantia nigra pars compacta; PD, Parkinson’s disease.

Sixteen 8-week-old male C57BL/6J mice (23 ± 2 g) were purchased from Beijing Vital River Laboratory (Beijing, China) and housed in an air-conditioned room at 22 ± 2°C with 55% ± 5% relative humidity and a 12 h light/dark cycle. The experiment was conducted according to the guidelines approved by the Ethical Committee of Experimental Animal Care (approval no: 180703) at The Hong Kong Polytechnic University Shenzhen Institute and all efforts were made to minimize animal suffering or discomforts. The mice were given a standard laboratory rodent diet (AIN-93M) and provided free access to distilled water. Animal experiment started on Monday and the PD mice model was achieved by i.p. injection of MPTP (20 mg/kg) twice a week in the morning (Wednesday and Saturday) for 5 weeks (Model group); while normal mice were injected with the same volume of saline twice a week for 5 weeks (Normal group). All the mice had adapted to the environment for 1 week before the start of the experiment. The animal experimental timeline is shown in Figure 1A.

### Grip Strength Test

The grip strength of both the forelimb and hindlimb (4 paws) was measured using a grip strength meter (An Hui Zheng Hua Biologic Apparatus Facilities Ltd., Co., China). The grip strength of each mouse was measured 3 times with a 3 min interval between each measurement to prevent fatigue of animals. Individual muscular function was assessed by sensing the peak amount of force for each measurement.

### Open Field Test

The open field test is another frequently used behavior test to assess spontaneous locomotor activity in PD models (Yan et al., 2018). The apparatus is a chamber consisting of a square arena with a surrounding wall; and it is connected to a video tracking system (SMART 3.0 Panlab Harvard Apparatus Instruments, Inc., United States). In the field (40 cm ^∗^ 40 cm), an area is appointed to be a target area (20 cm ^∗^ 20 cm) in the center of the field. Each mouse was placed in the center of the field, and their locomotor activity was recorded by the computer as total distance traveled (cm) and moving speed (cm/sec) in the target area within a 5-min period. Furthermore, an activity index (cm^2^/s) was defined as the whole area crossed by mice in the 5-min period.

### Sample Collection

After the behavioral tests, the mice were fasted overnight then sacrificed. Fecal samples (*n* = 8) for 16S rRNA gene sequencing were taken from the proximal colon, approximately one inch downstream of the cecum. Colon contents were collected by elevating one end of the large intestine and pushing contents into a sterile sample container. After rinsing with saline, colon tissues (*n* = 8) were collected and stored at −80°C. Brain tissues of the substantia nigra pars compacta (SNpc) and striatum (*n* = 6) were separated and stored at −80°C for further measurements. The whole brain (*n* = 2) was removed for immunohistochemistry staining.

### Analysis of Fecal Short Chain Fatty Acids (SCFAs)

Mice fecal samples were thawed, homogenized, acidified and centrifuged. Fifty microliter internal standard (1% 2-ethyl butyrate acid solution) and 500 μl diethyl ether anhydrous were added into the supernatants and centrifuged again. One microliter of the upper diethyl ether layer was injected into a GC instrument (Agilent 7820A; Agilent Technologies, United States) for determination of SCFAs concentrations using a published method (David et al., 2014).

### Western-Blot Analysis

SNpc and colon tissues were collected and homogenized. Lysates were centrifuged and total proteins were acquired and separated by SDS-PAGE in gels. After separation, the proteins were transferred to PVDF membrane. The membranes were blocked with 5% non-fat milk followed by incubation with primary antibody of mouse anti-TH (1:1,000, Millipore, United States), rabbit anti-LC3B (1:1,000, Novus, United States), mouse anti-p62 (1:1,000, Santa Cruz, United States), mouse anti-Bax (1:1,000, CST, United States), mouse anti-Bcl-2 (1:1,000, Santa Cruz, United States), mouse anti β-actin (1:1,000, Santa Cruz, United States) and secondary antibody of goat-anti-mouse IgG (1:1,000, Santa Cruz, United States) or goat-anti-rabbit IgG (1:1,000, Beyotime Biotechnology, China). Bands were visualized by using a chemiluminescence kit under the ECL system. Densitometry was performed by using Image J software.

### Measurement of Striatal Dopamine (DA) Levels by LC-MS

0.1 g of each striatum sample was put into a centrifuge tube and diluted with 500 μl distilled and deionized water, followed by homogenization. Acetonitrile was added into the homogenate, and the mixture was centrifuged at 14,000 g/min for 10 min. Then, supernatants were collected, blow dried with nitrogen, and finally dissolved in mobile phase. An ultra-high performance liquid phase system (Waters, MA, United States) that included a binary pump, an online degasser, an autosampler system and a column oven was used in chromatographic analysis. Standards of DA were freshly prepared and injected to the column for calibration.

### Immunohistochemistry (IHC) Staining of TH-Immunoreactive (TH-IR) Neurons

The brain and colon were post fixed in 4% paraformaldehyde at 4°C overnight and immersed in different concentrations of sucrose for dehydration. Frozen coronal sections (16 μm) were serially cut through the SNpc or the colon with a freezing cryostat microtome (CM1950, Leica, Germany). Sections were then attached to slides and treated with 1% bovine serum to block non-specific binding sites followed by incubation with mouse anti TH (1:1,000, Millipore, United States) and secondary antibodies (goat anti-mouse IgG, 1:1,000, Santa Cruz, United States). TH-IR neurons in the SNpc or the colon were visualized by 3′-diaminobenzidine (DAB) staining.

### 16S rRNA Gene Sequencing and Data Analysis

The proximal colon content DNA was extracted by QIAamp DNA stool kit (Qiagen, United States) with a previously described protocol (Lacombe et al., 2013). The extracted DNA from each sample was used as a template to amplify the V3-V4 region of 16S rRNA genes. PCR amplification, pyrosequencing of the PCR amplicons, and quality control of raw data were performed as described in previously published paper (Liu et al., 2017). The sequence data were preprocessed using MiSeq Control Software v2.4.1. 16S rRNA gene sequences were analyzed by QIIME pipeline (v1.8.0). GreenGene database (v201305) was used for sequence alignment and taxonomy assignment (greengenes.lbl.gov). All raw sequence data have been deposited to the China National GeneBank (CNGB) with project accession number: CNP0001553.

### Statistical Analysis

All values are expressed as mean ± standard error of the mean (SEM). Subsequent statistical analyses were performed using Graphpad Prism version 5.0 (Graphpad, United States). Unpaired *T*-test was performed between two groups. Differences in *p-*values of less than 0.05 were considered statistically significant. In addition, the linear discriminant analysis (LDA) effect size (LEFSe) method was used to test significant differences of microbiome features between two groups; and the cutoff value is the absolute log_10_ LDA score > 2.0.

## Results

### Colonic Dopaminergic Neurons Changed Reversely With Those in the Midbrain of PD Mice

The pole test, open filed test and grip strength test were employed to evaluate the locomotor deficits of mice. Fecal discharge frequency test was performed to appraise gastrointestinal (GI) function. As expected, the muscle strength of PD mice declined greatly (*p* < 0.05 vs. Normal; Figure 1B), suggesting the destroyed motor control and coordination by chronic MPTP injection in mice. In the open field test, PD mice (vs. Normal) exhibited locomotor deficits with reduced walking distance (*p* < 0.05; Figure 1C) and activity (*p* < 0.05; Figure 1D). No statistically significant changes were found in PD mice in the pole test and GI function test (Supplementary Figure 1). Accordingly, TH (a marker of dopaminergic neurons) protein expressions (Figure 1G and Supplementary Figure 2A) and its immunohistochemistry (IHC) staining (Figure 1E) in the SNpc of PD mice declined greatly (*p* < 0.01 vs. Normal); and DA content in the striatum (*p* < 0.05 vs. Normal; Table 1) reduced significantly in such a chronic PD mice model. Conversely, our results demonstrated that TH protein expressions (*p* < 0.01; Figure 1H and Supplementary Figure 2B), IHC staining of TH (Figure 1F) and DA content (*p* < 0.05; Table 1) were highly elevated in the colon of PD mice (vs. Normal). Opposite changes of dopaminergic neurons were found in the midbrain and the colon of mice in response to chronic MPTP injection.

**TABLE 1:** Striatal and colonic dopamine (DA) content and fecal short chain fatty acids (SCFAs) levels in normal and model mice of Parkinson’s disease (PD)^*a,b*^.

**Groups**	**DA Content (ng/mg tissues)**		**Fecal SCFAs (nmol/g wet feces)**			
	**Striatum**	**Colon**	**Total SCFAs**	**Acetate**	**Propionate**	**Butyrate**
Normal	6.26 ± 0.73	0.06 ± 0.02	5.85 ± 0.94	2.28 ± 0.28	2.06 ± 0.39	0.72 ± 0.11
Model	3.99 ± 0.52*	0.30 ± 0.11*	2.62 ± 0.37**	0.90 ± 0.17***	1.03 ± 0.10*	0.69 ± 0.10

^*a*^Values are expressed as mean ± SEM, n = 6–8. *p < 0.05, **p < 0.01, ***p < 0.001 vs. Normal group.

*^*b*^DA, dopamine; SCFAs, short chain fatty acids; PD, Parkinson’s disease.*

### Autophagy and Apoptosis Changed Reversely in the Colon With the Midbrain in PD Mice

During autophagy, protein light chain 3 (LC3I) is conjugated to phosphatidylethanolamine to form LC3-II, which results in the recruitment of ubiquitinated p62/SQSTM1 (p62) attached to the cargo molecules destined for degradation (Yoshii and Mizushima, 2017). Higher turnover of LC3II to LC3I (LC3 conversion) accompanied by low levels of p62 in cells could reflect stimulated autophagic activity. Similarly, Bax protein acts as a pro-apoptotic member, but Bcl-2 protein is an anti-apoptotic member; Bax and Bcl-2 expressions and their ratios are markers of apoptosis (Antonsson, 2001).

**FIGURE 2:**
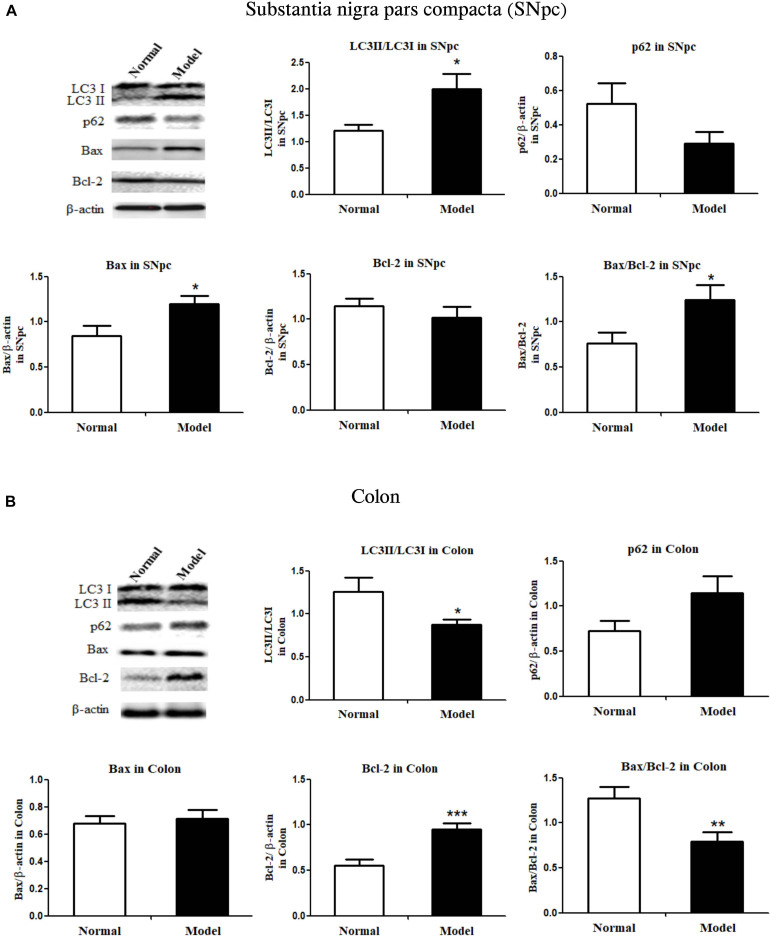
Critical protein expressions in autophagy and apoptosis in both the substantia nigra pars compacta and colon of mice. Protein expressions of LC3I, LC3II, p62, Bax, Bcl-2 in SNpc **(A)** and colon **(B)** in normal and PD model mice (one representative band for target protein and endogenous β-actin from each group; and the following bar chart (*n* = 6–8) shows the ratio of target protein/β-actin or ratio of LC3II/LC3I and Bax/Bcl-2 in both SNpc and colon of mice). Values are expressed as mean ± SEM. *p < 0.05, **p < 0.01, ***p < 0.001 vs. Model group. SNpc, substantia nigra pars compacta; PD, Parkinson’s disease.

As shown in Figure 2A and Supplementary Figure 3A, LC3II/LC3I ratio (LC3 conversion) was significantly enhanced (*p* < 0.05 vs. Normal), while p62 protein expression was seemingly downregulated in the SNpc of PD mice, indicating the stimulated autophagy in the SNpc of PD mice. Conversely, LC3II/LC3I ratio was apparently repressed (*p* < 0.05 vs. Normal), and p62 expression appeared to have increased in the colon of PD mice, suggesting inhibited autophagy in the colon of PD mice. Figure 2B and Supplementary Figure 3B, suggesting inhibited autophagy in the colon of PD mice.

Bax proteins were found to increase (*p* < 0.05), while Bcl-2 did not change much in the SNpc of PD mice; and Bax/Bcl-2 ratio increased significantly (*p* < 0.05) in the SNpc of PD mice (vs. Normal; Figure 2A and Supplementary Figure 4A). Colonic Bax proteins were not influenced while colonic Bcl-2 protein expression was significantly increased (*p* < 0.001) in response to chronic MPTP injection, the ratio of Bax/Bcl-2 in the colon was greatly lowered (*p* < 0.01) in PD mice (vs. Normal; Figure 2B and Supplementary Figure 4B). The results demonstrated autophagy and apoptosis were enhanced in the SNpc, but inhibited in the colon of PD mice.

### Fecal SCFAs Production Declined in PD Mice

Chronic MPTP injection resulted in significant decline of the total fecal concentrations of SCFAs in mice (*p* < 0.01 vs. Normal; Table 2). PD mice had significantly lower concentrations of acetate (61% decline; *p* < 0.001) and propionate (50% decline; *p* < 0.05) in feces (vs. Normal), while fecal butyrate concentrations in PD and normal mice were equal.

**TABLE 2:** Relative abundance of phylum, class, order, family, genus > 1% in normal and PD model mice^*a*^.

**Groups**		**Phylum**			**Class**	
		***Bacteroidetes***	***Firmicutes***		***Bacteroidia***	***Erysipelotrichia***
Normal		70.35 ± 1.79	26.32 ± 1.66		70.35 ± 1.79	1.66 ± 0.42
Model		60.57 ± 3.21*	34.91 ± 2.76*		60.56 ± 3.21*	2.97 ± 0.52*

**Groups**	**Order**		**Family**			**Genus**
	***Bacteroidales***	***Erysipelotrichales***	***S24_7***	***Ruminococcaceae***	***Erysipelotrichaceae***	***Allobaculum***

Normal	70.35 ± 1.79	1.66 ± 0.42	58.62 ± 2.03	5.27 ± 0.45	1.60 ± 0.42	1.53 ± 0.41
Model	60.56 ± 3.21*	2.97 ± 0.52*	48.26 ± 3.07**	6.81 ± 0.53*	2.97 ± 0.52*	2.81 ± 0.52 *

^*a*^Values are expressed as mean ± SEM, n = 8. *p < 0.05, **p < 0.01 vs. Normal group.

### Significant Changes of Microbial Compositions in PD Mice

**FIGURE 3:**
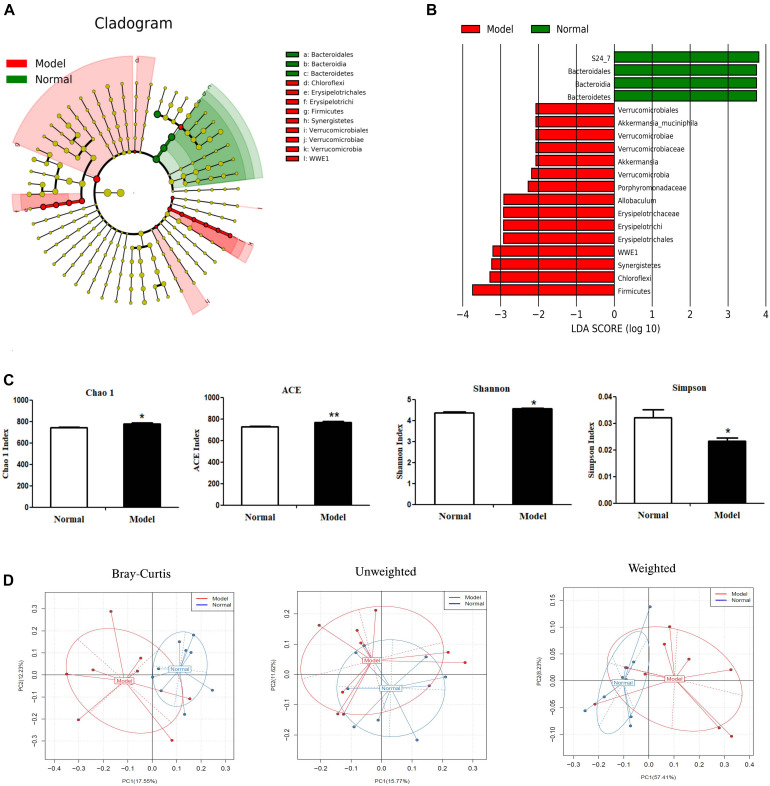
Microbial composition analysis by 16S rRNA gene sequencing. **(A)** Cladograms displaying the taxa with significantly different abundance. Model 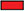 vs. 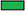 Normal group. Only the taxa with absolute log_10_ LDA scores > 2.0 are displayed. **(B)** Selected microbial taxa significantly impacted in PD mice. Only those taxa with the absolute log_10_ LDA scores > 2.0 are listed in the charts. **(C)** α-diversity as indicated by microbial diversity indices of Chao1, ACE, Shannon, Simpson. **(D)** β-diversity analysis: Principal coordinates analysis (PCoA) derived from Bray-Curtis distances, unweighted and weighted UniFrac among samples of two groups (Model 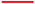 vs. Normal 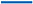). *p < 0.05, **p < 0.01 vs. Model group.

Alpha diversity analysis (Figure 3C) showed that microbiota in PD model mice had higher values of Chao1 (*p* < 0.05), ACE (*p* < 0.01), and Shannon (*p* < 0.05), but lower values of Simpson (*p* < 0.05) (vs. Normal). In beta diversity analysis (Figure 3D), principal coordinates analysis (PCoA) derived from Bray-Curtis distances, unweighted and weighted UniFrac revealed there were no significant differences between model and normal groups. These results suggested chronic MPTP injection resulted in higher microbial community richness and diversity in PD mice, but PD mice had no significant contrast of microbial communities compared with normal mice.

As shown in Figures 3A,B and Table 2, chronic MPTP injection significantly decreased the abundance of the phylum *Bacteroidetes* (*p* < 0.05), but increased the abundance of the phylum *Firmicutes* (*p* < 0.05). Accordingly, the abundance of *Bacteroidia* (Class) (*p* < 0.05), *Bacteroidales* (Order) (*p* < 0.05), *S24_7* (Family) (*p* < 0.01) under the phylum *Bacteroidetes* decreased greatly in PD mice (vs. Normal); while the abundance of *Erysipelotrichia* (Class) (*p* < 0.05), *Erysipelotrichales* (Order) (*p* < 0.05), *Erysipelotrichaceae* (Family) (*p* < 0.05), *Ruminococcaceae* (Family) (*p* < 0.05), *Allobaculum* (Genus) (*p* < 0.05) under the phylum *Firmicutes* increased significantly in PD mice (vs. Normal). Some other species also exhibited significantly higher abundance in PD mice compared with normal mice, including the phyla *Chloroflexi*, WWE1, and *Verrucomicrobia*, but their relative abundance was extremely low (Figures 3A,B). These results suggested chronic MPTP injection resulted in gut microbiota (GM) dysbiosis in PD mice.

## Discussion

The role that the gut-brain-microbiota axis plays in the pathogenesis of PD has become a research hotspot, but the detailed mechanisms are still unclear. Appropriate animal model to study gut-brain axis in PD were not confirmed. Our results demonstrated such a classical chronic PD model mice exhibited pathological hallmarks of PD in mice brain as indicated by apparent locomotor deficits and nigral dopaminergic neuronal loss. However, colonic dopaminergic neurons were increased and GI dysfunction test did not show any changes in PD mice compared with normal mice. Limited researches investigated effects of MPTP on dopaminergic neurons in ENS. Results from two animal studies demonstrated acute injection of MPTP in 1 day led to loss of dopaminergic neurons in mice ileum (Ellett et al., 2016; Poirier et al., 2016). One study by using chronic MPTP injection (same protocol as that in the present study) showed the increase of TH expressions in mice ileum which was contradictory with those in mice midbrain (Lai et al., 2018). Such result in this study is consistent with our finding, although the animal sample collection time and detected intestinal segment are different. Relevant information was all included in Supplementary Table 1, and we can see that different MPTP injection protocols might lead to different changes of enteric dopaminergic neurons.

Apoptosis and autophagy are two major machineries of the degeneration of dopaminergic neurons and their imbalance accelerates PD progression (Ghavami et al., 2014; Liu et al., 2019). MPTP was reported to induce excessive autophagy and apoptosis in the CNS (Li et al., 2011; Wang et al., 2020), but there weren’t any reports on its actions on autophagy and apoptosis in the ENS. Our results showed autophagy activity (higher LC3 conversion) and apoptosis (higher Bax levels) were stimulated in mice SNpc, but inhibited (lower LC3 conversion and higher Bcl-2 levels) in mice colon in response to chronic MPTP injection for 5 weeks. These results implied opposite changes of autophagy and apoptosis in the SNpc and colon determined the reversed changes of dopaminergic neurons in the CNS and ENS in such a chronic MPTP model.

As reported, PD patients exhibited GM dysbiosis and lower production of fecal SCFAs (Unger et al., 2016). Our results indicated chronic MPTP injection resulted in GM dysbiosis in PD mice. Several clinical studies reported that the GM in PD patients displayed higher microbial community richness and diversity indicated by alpha diversity indexes in comparison with matched healthy controls (Keshavarzian et al., 2015; Qian et al., 2018; Lin et al., 2019). Consistently, in the present study, chronic MPTP injection resulted in higher values of alpha diversity indexes including Chao1, ACE and Shannon that were positively related to microbial richness and diversity, and lower values of Simpson that was inversely associated with microbial richness and evenness. However, the diverse results of the gut microbiome in PD patients may make it hard to compare the present results in mice with those in humans. And it was found different MPTP injection protocols and even different collection time of animal samples after MPTP injection could lead to various alterations of GM compositions and SCFAs production (Supplementary Table 1). The mechanism for MPTP to regulate GM compositions is yet to be elucidated. Recent studies presented bacterial phages (known as gut phagobiota) might also be implicated in regulating GM during neurodegenerative disease including PD (Tetz et al., 2018; Tetz and Tetz, 2018). GM and gut phagobiota interactively involved in PD pathogenesis, which might be one of the possible directions to disclose GM dysbiosis mechanisms in either PD patients or animal models.

The relationship of GM and autophagy is a novel topic in recent years. Although the detailed mechanism is not fully clarified, most relevant findings were disclosed in mechanism studies of IBD. Defective autophagy was suggested to have a strong impact on IBD pathogenesis, via disruption of intestinal barrier integrity, affecting GM composition and amplifying intestinal inflammation (Larabi et al., 2020). Furthermore, GM and autophagy influence each other and their interplay help to maintain intestinal homeostasis (Kim et al., 2020; Larabi et al., 2020). Until now, no study has reported the function of interplay of GM with autophagy during PD pathogenesis. In our present study, MPTP was found to influence both GM compositions and autophagy activity simultaneously, and it can be speculated their interplay finally resulted in GM dysbiosis and defective autophagy in the colon of PD mice.

Short chain fatty acids (SCFAs) (mainly including acetate, propionate and butyrate) are the main microbiota metabolites produced in the colon (Miller and Wolin, 1996). Acetate and propionate are mainly produced by *Bacteroidetes*, whereas *Firmicutes* are the primary contributors of butyrate (Feng et al., 2018), the significantly lower fecal acetate and propionate levels in our study might be attributed to the decreased abundance of *Bacteroidetes*; while no decrease in fecal butyrate concentration could be partially due to the simultaneously increased *Firmicutes* abundance in PD mice. Acetate (Xu et al., 2019) and butyrate (Zhou et al., 2020) were found to stimulate autophagy in normal intestinal epithelial cells. Propionate was reported to activate autophagy in colon cancer cells via decreased mTOR activity and enhanced AMP kinase activity (Tang et al., 2011). It suggested lower production of fecal SCFAs derived by GM dysbiosis, acetate, and propionate in particular, might be one of the mediators of the downregulated autophagy in the colon of PD mice. Accordingly, the dysfunctionally inhibited autophagy could explain the reduced apoptosis in the colon of PD mice.

The scope of the present study is limited to observe end-point changes of dopaminergic neurons in both the SNpc and the colon from a classical PD mice model made by chronic MPTP injection for 5 weeks, followed by mechanism investigations to reveal the relationships of enteric dopaminergic neurons with autophagy, apoptosis and GM. Our findings were absent in the published information and would be helpful to fill a knowledge gap. However, our mechanism studies might not be very comprehensive, and be limited by the complexity of too many factors involved. Further studies are required to disclose how GM and autophagy interact in regulating enteric dopaminergic neurons; and how gut phagobiota interactively with GM involve in PD pathogenesis.

## Conclusion

In conclusion, our results showed opposite changes of dopaminergic neurons in the colon and in the midbrain in a classical PD model established by chronic MPTP injection. PD mice exhibited apparent locomotor deficits and nigral dopaminergic neuronal loss in the CNS, but there were opposite changes of colonic dopaminergic neurons in the ENS. Mechanism investigation implied autophagy activity and apoptosis were stimulated in the SNpc, but inhibited in the colon of PD mice, which contributed to the opposite changes of dopaminergic neurons in CNS and colonic ENS. Chronic MPTP injection influenced GM compositions and autophagy activity simultaneously, and their interplay finally resulted in GM dysbiosis and defective autophagy in the colon of PD mice. Furthermore, GM dysbiosis derived fecal SCFAs, acetate and propionate in particular, might be one of the mediators of the downregulated autophagy in the colon of PD mice. Although alterations of GM composition and SCFAs production in such a chronic PD mice model may, to some extent, mimic those in PD patients, intestinal pathology found in this PD mice showed reversed changes with those in the brain. Such a chronic PD mice model might not be an ideal model to study role of gut-brain axis in PD progression. It is hope this study could provide more evidences to researchers in selection of animal models for PD studies.

## Data Availability Statement

All raw sequence data have been deposited to the China National GeneBank (CNGB) with project accession number: CNP0001553.

## Ethics Statement

The experiment was conducted according to the guidelines approved by the Ethical Committee of Experimental Animal Care (approval no: 180703) at the Hong Kong Polytechnic University Shenzhen Institute.

## Author Contributions

XL and XW performed the animal experiments. Z-RD detected most of the parameters. K-HL and C-HC helped to reorganize data and revise the manuscript. FZ helped to conduct analysis for 16S rRNA gene sequencing. XC, QZ, and W-TW helped to revise the manuscript. K-HW supervised the study and revised the manuscript. X-LD designed the study and wrote the manuscript. All authors contributed to the article and approved the submitted version.

## Conflict of Interest

FZ was employed by company BGI-Qingdao. The remaining authors declare that the research was conducted in the absence of any commercial or financial relationships that could be construed as a potential conflict of interest.
