# Supplementary material for: Colonic Dopaminergic Neurons Changed Reversely With Those in the Midbrain via Gut Microbiota-Mediated Autophagy in a Chronic Parkinson’s Disease Mice Model
Source: Front Aging Neurosci. 2021 Apr 12;13:649627. doi: 10.3389/fnagi.2021.649627 (PMC8071868; doi:10.3389/fnagi.2021.649627)
Supplement: Supplementary file 1 [file Data_Sheet_1.docx]

**Supplemental Information**

**Colonic dopaminergic neurons changed reversely with those in the midbrain via gut microbiota-mediated autophagy in a chronic Parkinson’s disease mice model**

Xin Liu^1,2,3^, Zhong-Rui Du^1,2,3,6^, Xiong Wang^1^, Kar-Him Luk^1,2,3^, Cheuk-Hin Chan^1,2,3^, Xu Cao^4,5^, Qing Zhao^7^, Fang Zhao^8^, Wing-Tak Wong^1,2,3^, Ka-Hing Wong^1,2,3^* and Xiao-Li Dong^1,2,3^*

**1. Supplemental Methods:**

**1.1 Pole test**

As described in previous studies [1], mice were positioned head up at the top of a vertical rough-surfaced pole in order to evaluate bradykinesia in the mice. The time each mouse took to turn and reach the floor was recorded. Two days before testing, each mouse was trained, and on the test day, mice were allowed to practice five times and then tested three times, with each trial lasted for a maximum of 3 min.

**1.2 Gastrointestinal function tests (Fecal discharge frequency)**

In the gastrointestinal function tests, the mice were fasted overnight. The next day, the mice were given access to food for 2h prior to testing, and then put in clean isolated cages to observe mice fecal discharge frequency. Fecal pellets per mouse were recorded within different time periods, from 5min to 20min.

**2. Supplemental Figures**

**2.1 Results form pole test and gastrointestinal function test**

A

B C

**Supplemental Figure 1 (Fig. S1)**. Pole tests and fecal discharge frequency in normal and model mice of Parkinson’s disease (PD). (A) Total time to descend (s); (B) Fecal pellets per mouse at four time points; (C) Total fecal pellets within 20 mins.

**2.2 Original images of western blot (WB) bands for tyrosine hydroxylase and β-actin**

**A Substantia nigra pars compacta (SNpc)**

**
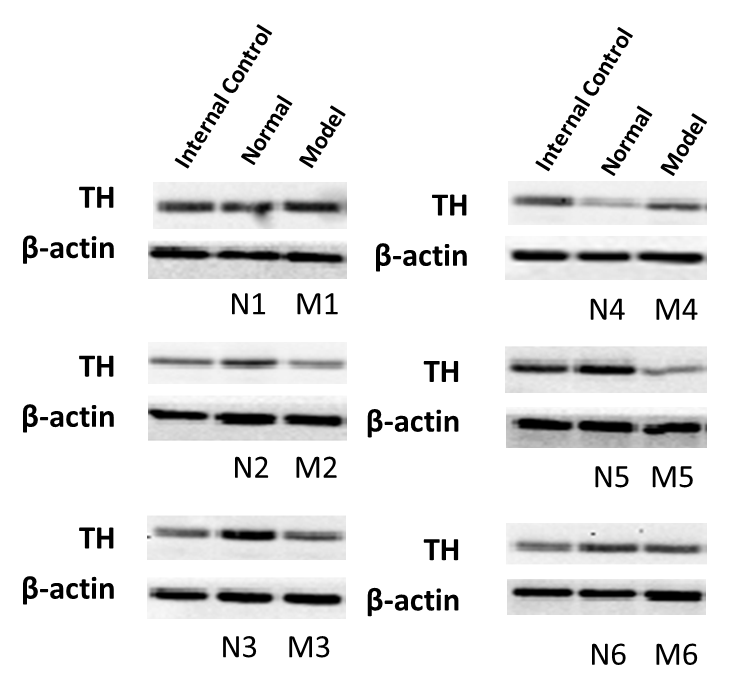
**

**B Colon**

**
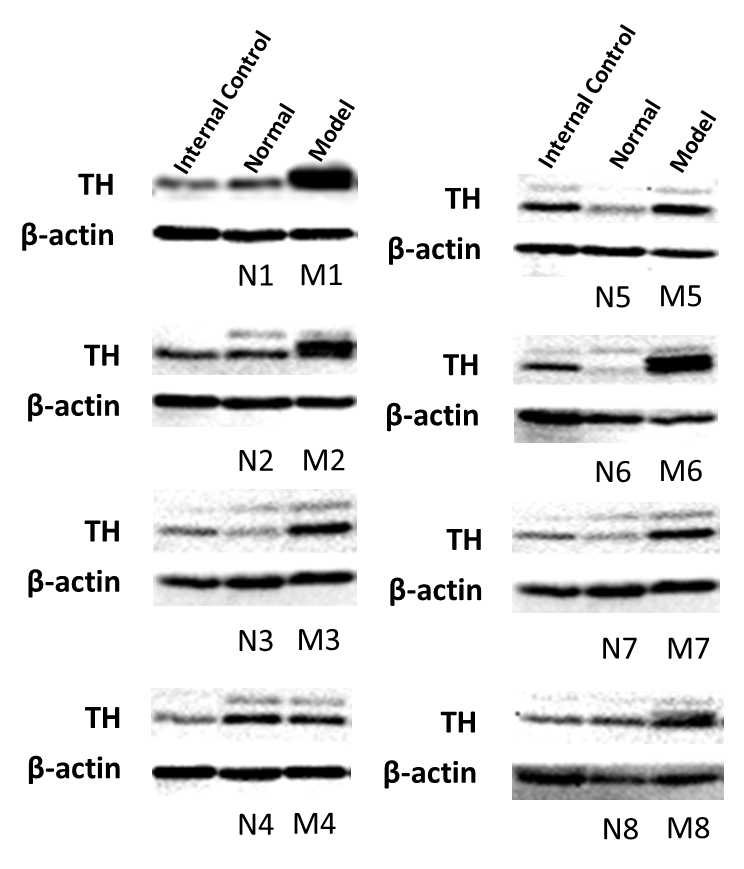
**

**Supplemental Figure 2 (Fig. S2)**. Original images of western blot (WB) bands to display protein expressions of tyrosine hydroxylase (TH) and its endogenous control (β-actin) in the substantia nigra pars compacta (SNpc) (A) and the colon (B) in each mouse from either Normal or PD Model group. Not all protein samples were run in the same gels and one mixed protein sample as internal control for inter membrane comparisons. N, Normal; M, Model; PD, Parkinson’s disease

**2.3 Original images of WB bands for critical proteins in autophagy and β-actin**

**A1 SNpc A2 SNpc**

**
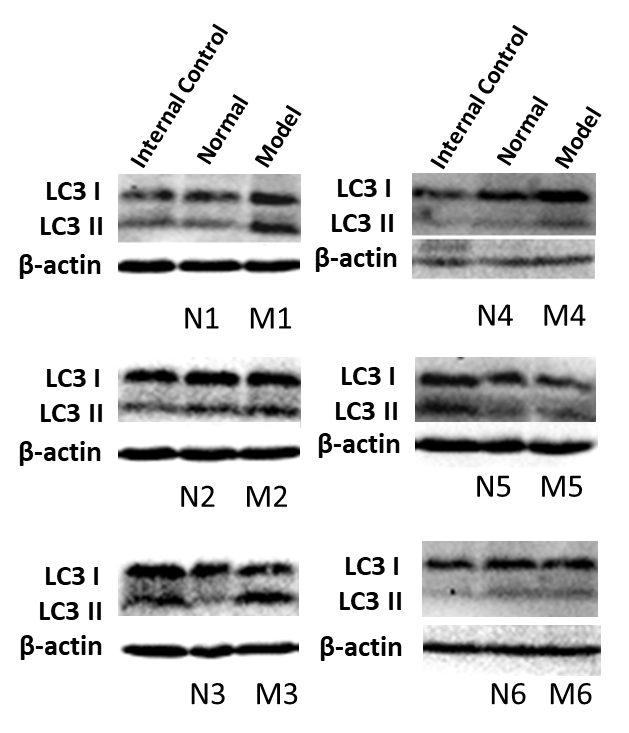

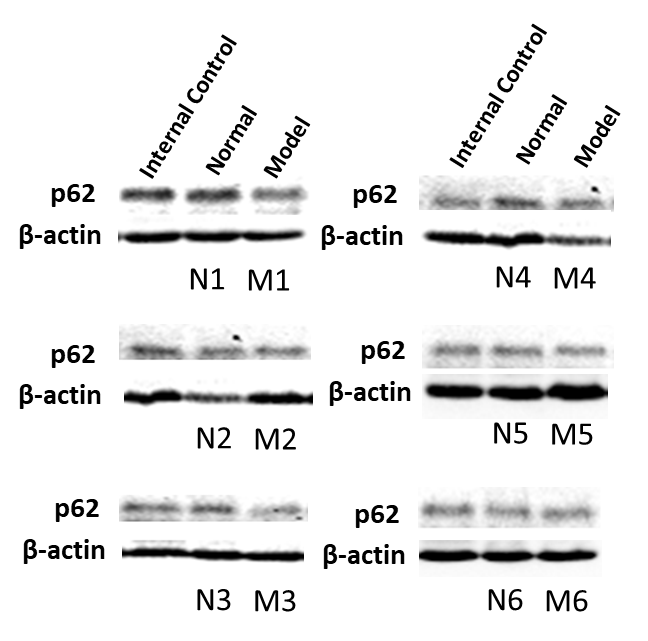
**

**B1 Colon B2 Colon**

**
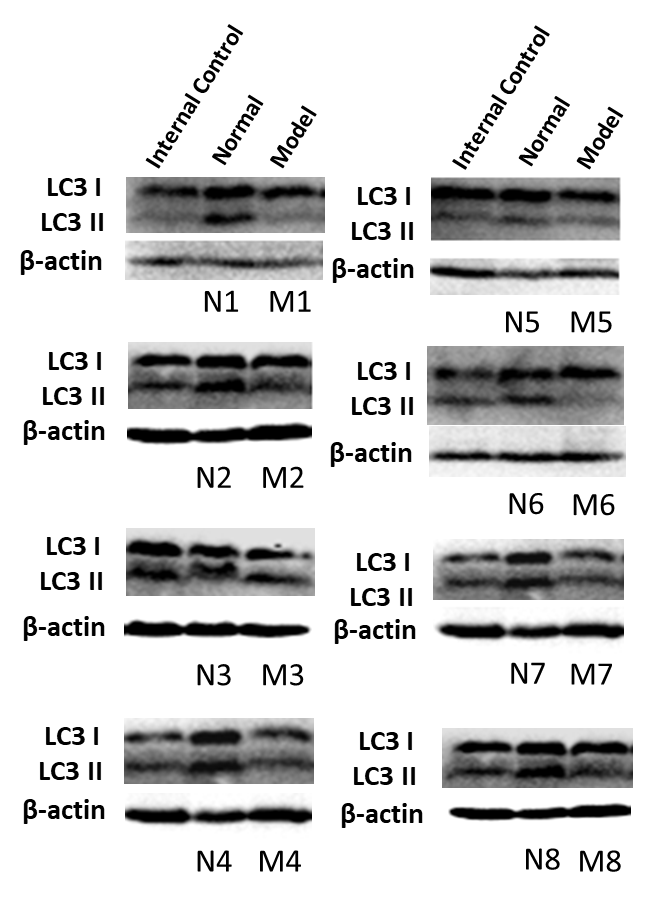

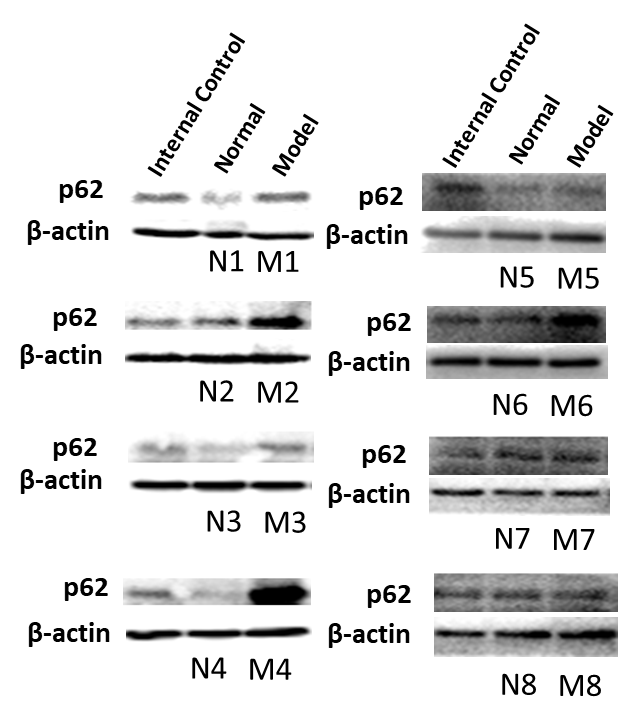
**

**Supplemental Figure 3 (Fig. S3)**. Original images of WB bands to display protein expressions of LC3 I and LC3 II (A1, B1), p62 (A2, B2) and its endogenous control (β-actin) in the substantia nigra pars compacta (SNpc) (A) and the colon (B) in each mouse from either Normal or PD Model group. Not all protein samples were run in the same gels and one mixed protein sample as internal control for inter membrane comparisons. N, Normal; M, Model; PD, Parkinson’s disease

**2.4 Original images of WB bands for critical proteins in apotosis and β-actin**

**A1 SNpc A2 SNpc**


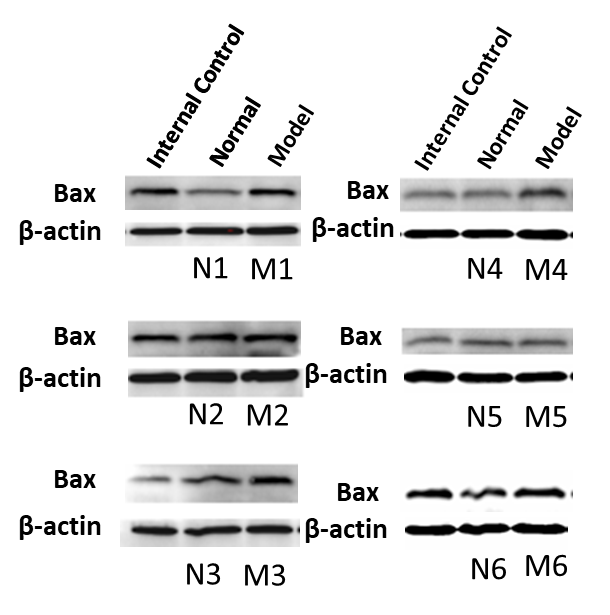

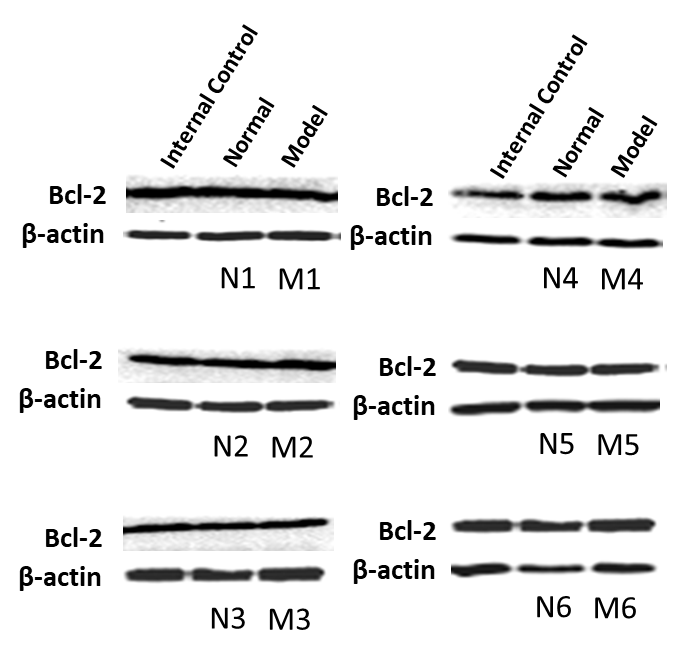


**B1 Colon B2 Colon**


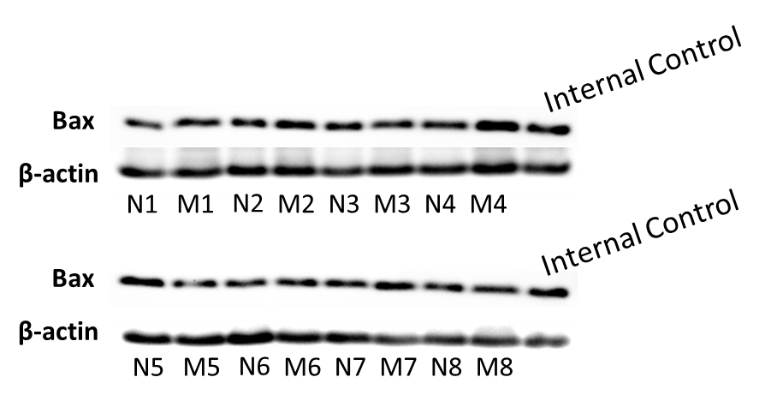

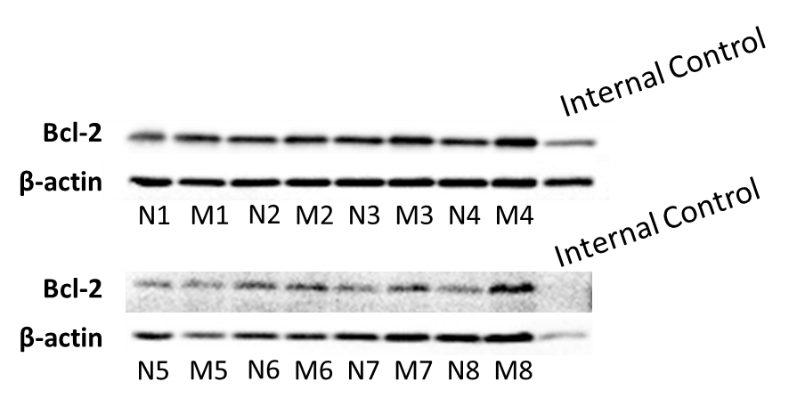


**Supplemental Figure 4 (Fig. S4)**. Original images of WB bands to display protein expressions of Bax (A1, B1), Bcl-2 (A2, B2) and its endogenous control (β-actin) in the substantia nigra pars compacta (SNpc) (A) and the colon (B) in each mouse from either Normal or PD Model group. Not all protein samples were run in the same gels and one mixed protein sample as internal control for inter membrane comparisons. N, Normal; M, Model; PD, Parkinson’s disease

**3. Supplemental Table 1** Summary of changes in GM composition, fecal SCFAs production and enteric dopaminergic neurons in response to different MPTP injection protocols and time of animal sample collection ^1,2^

|  | **MPTP injection protocols** | **Time of animal sample collection** | **GM composition changes**  **in PD mice by 16S rRNA gene sequencing** | **Fecal SCFAs production in PD mice** | **Enteric dopaminergic neuron changes in PD mice** |
| --- | --- | --- | --- | --- | --- |
| **Acute PD mice model** | MPTP (20 mg/kg) i.p. injection, 4 times a day with interval of 2 hours [2] | 8^th^ day after MPTP injection | α-diversity:↑  Phylum: *Firmicutes*↑, *Tenericutes*↑; *Proteobacteria*↓  Genus: *Ruminococcus*↑*, Oscillospira*↑*, Turicibacter*↑*, Clostridium*↑*,*  *Anaerostipes*↑*, Dehalobacterium*↑*, and Holdemania*↑; *Blautia*↓ | Acetic acid: –  Propanoic acid: ↓  Butyric acid: ↑ | Not detect |
|  | MPTP (15 mg/kg) i.p. injection, 4 times a day with interval of 2 hours [3] | 4^th^ day after MPTP injection | α-diversity and β-diversity: −  Order: *Turicibacterales*↑*;*  Family: *Turicibacteraceae*↑*, Odoribacteraceae*↑*, Prevotellaceae*↑*;*  Genus: *Turiciacter, Odoribacter*↑*, Lactobacillus*↑ | Acetic acid: –  Propanoic acid: –  Butyric acid: ↓ | Not detect |
|  | MPTP (15 mg/kg) i.p. injection, 4 times a day with interval of 2 hours [4] | 6^th^ day after MPTP injection | Not detect | Not detect | Ileum TH expression by IHC staining: ↓ |
|  | MPTP (10 mg/kg) i.p. injection, 4 times a day with interval of 2 hours [5] | 22^nd^ day after MPTP injection | Not detect | Not detect | Ileum TH expression by immunofluorescence staining: ↓ |
| **Sub-acute PD mice model** | MPTP (30 mg/kg) i.p. injection, once daily for continous 5 days [6] | 6^th^ day after the last MPTP injection | α-diversity and β-diversity: −  Family: *Bifidobacteriaceae*↑ | Acetic acid: ↓  Propanoic acid: ↓  Butyric acid: ↓ | Not detect |
|  | MPTP (30 mg/kg) i.p. injection, once daily for continous 5 days [7] | 14^th^ day after the last MPTP injection | α-diversity and β-diversity: −  Phylum: *Proteobacteria*↑*; Firmicutes*↓  Order: *Turiciacterales*↑*, Enterobacterales*↑*, Bacteroidales*↑*; Clostridia*↓ | Acetic acid: ↑  Propanoic acid: ↑  Butyric acid: ↑ | Not detect |
| **Chronic PD mice model** | MPTP (18 mg/kg) i.p. injection, twice a week in  the afternoon (Tuesday and Saturday) for 5 weeks [8] | 2^nd^ day after the last MPTP injection | α-diversity:↑  Phylum: *Proteobacteria*↓  Family: *Prevotellaceae*↑ | Not detect | Ileum DA content: ↓  Ileum TH expression: ↑ |
|  |  | 22^nd^ day after the last MPTP injection | α-diversity: −  Phylum: *Proteobacteria*↓  Order: *Erysipelotrichales*↑*; Clostridiales*↓  Family: *Erysipelotrichaceae*↑*, Lachnospiraceae*↓ |  |  |
|  | MPTP (20 mg/kg) i.p. injection, twice a week in  the morning (Wednesday and Saturday) for 5 weeks  (our present study) | 38^th^ day after the last MPTP injection | α-diversity:↑ β-diversity: −  Phylum: *Firmicutes*↑*; Bacteroidetes*↓  Class: *Erysipelotrichia*↑*; Bacteroidia*↓  Order: *Erysipelotrichales*↑*; Bacteroidales*↓  Family: *Erysipelotrichaceae*↑*, Ruminococcaceae*↑*; S24_7*↓  Genus: *Allobaculum*↑ | Acetic acid: ↓  Propanoic acid: ↓  Butyric acid: − | Colonic DA content:↑  Colonic TH expression: ↑ |

Note: ^1^Abbreviations used: GM, gut microbiota; SCFAs, short chain fatty acids; MPTP, 1-methyl-4-phenyl-1,2,3,6-tetrahydropyridine; PD, parkinson’s diseaes; DA, dopamine; TH, tyrosine hydroxylase ; ^2^↑refers to upregulation; ↓refers to downregulation.

**References:**

[1] D. Huang, Z. Wang, J. Tong, M. Wang, J. Wang, J. Xu, X. Bai, H. Li, Y. Huang, Y. Wu, Y. Ma, M. Yu, and F. Huang, Long-term Changes in the Nigrostriatal Pathway in the MPTP Mouse Model of Parkinson's Disease. Neuroscience 369 (2018) 303-313.

[2] Z.L. Zhou, X.B. Jia, M.F. Sun, Y.L. Zhu, C.M. Qiao, B.P. Zhang, L.P. Zhao, Q. Yang, C. Cui, X. Chen, and Y.Q. Shen, Neuroprotection of Fasting Mimicking Diet on MPTP-Induced Parkinson's Disease Mice via Gut Microbiota and Metabolites. Neurotherapeutics 16 (2019) 741-760.

[3] X.L. Dong, X. Wang, F. Liu, X. Liu, Z.R. Du, R.W. Li, C.H. Xue, K.H. Wong, W.T. Wong, Q. Zhao, and Q.J. Tang, Polymannuronic acid prevents dopaminergic neuronal loss via brain-gut-microbiota axis in Parkinson's disease model. International journal of biological macromolecules 164 (2020) 994-1005.

[4] A.A. Poirier, M. Côté, M. Bourque, M. Morissette, T. Di Paolo, and D. Soulet, Neuroprotective and immunomodulatory effects of raloxifene in the myenteric plexus of a mouse model of Parkinson's disease. Neurobiology of aging 48 (2016) 61-71.

[5] L.J. Ellett, L.W. Hung, R. Munckton, N.A. Sherratt, J. Culvenor, A. Grubman, J.B. Furness, A.R. White, D.I. Finkelstein, K.J. Barnham, and V.A. Lawson, Restoration of intestinal function in an MPTP model of Parkinson's Disease. Scientific reports 6 (2016) 30269.

[6] J.F. Liao, Y.F. Cheng, S.T. You, W.C. Kuo, C.W. Huang, J.J. Chiou, C.C. Hsu, H.M. Hsieh-Li, S. Wang, and Y.C. Tsai, Lactobacillus plantarum PS128 alleviates neurodegenerative progression in 1-methyl-4-phenyl-1,2,3,6-tetrahydropyridine-induced mouse models of Parkinson's disease. Brain, behavior, and immunity 90 (2020) 26-46.

[7] M.F. Sun, Y.L. Zhu, Z.L. Zhou, X.B. Jia, Y.D. Xu, Q. Yang, C. Cui, and Y.Q. Shen, Neuroprotective effects of fecal microbiota transplantation on MPTP-induced Parkinson's disease mice: Gut microbiota, glial reaction and TLR4/TNF-α signaling pathway. Brain, behavior, and immunity 70 (2018) 48-60.

[8] F. Lai, R. Jiang, W. Xie, X. Liu, Y. Tang, H. Xiao, J. Gao, Y. Jia, and Q. Bai, Intestinal Pathology and Gut Microbiota Alterations in a Methyl-4-phenyl-1,2,3,6-tetrahydropyridine (MPTP) Mouse Model of Parkinson's Disease. Neurochemical research 43 (2018) 1986-1999.
